# Successful use of inhaled nitric oxide to decrease intracranial pressure in a patient with severe traumatic brain injury complicated by acute respiratory distress syndrome: a role for an anti-inflammatory mechanism?

**DOI:** 10.1186/1757-7241-17-5

**Published:** 2009-02-17

**Authors:** Thomas J Papadimos, Azedine Medhkour, Sooraj Yermal

**Affiliations:** 1 Department of Anesthesiology, College of Medicine, University of Toledo, 3000 Arlington Avenue, Toledo, OH 43614, USA; 2 Department of Anesthesiology, School of Medicine, University of Michigan, 1500 E. Medical Center Drive, Ann Arbor, MI 48109, USA; 3 Deparment of Surgery, Division of Neurosurgery, College of Medicine, University of Toledo, 3000 Arlington Avenue, Toledo, OH 43614, USA

## Abstract

Use of inhaled nitric oxide in humans with traumatic brain injury and acute respiratory distress syndrome has twice previously been reported to be beneficial. Here we report a third case. We propose that INO may decrease the inflammatory response in patients with increased intracranial pressure caused by traumatic brain injury accompanied by acute respiratory distress syndrome thereby contributing to improved outcomes.

## Background

Traumatic brain injury (TBI) affects 1.4 million Americans each year, which includes 1.1 million emergency department visits, 235,000 hospitalizations, and 50,000 deaths [1]. Approximately 5.3 million Americans are disabled with TBI [2] at a cost of $60 billion annually [3].

In the face of severe pulmonary insufficiency, such as occurs in neurogenic pulmonary edema, pneumonia, and acute lung injury (ALI)/acute respiratory distress syndrome (ARDS) oxygen delivery to the brain may be compromised. To avert an increase in intracranial pressure (ICP) caused by severe TBI, i.e., Glasgow Coma Scale (GCS ≤ 8), it has been recommended that the partial pressure of oxygen in arterial blood (PaO2) be maintained at a minimum of 100 mm Hg [4], cerebral perfusion pressure be maintained between 60–70 mm Hg [5], and the partial pressure of carbon dioxide in arterial blood (PaCO2) be maintained at 32–35 mm Hg [6].

The release of cytokines [7] and neuropeptides [8] that injure the brain occurs in patients subjected to TBI. This inflammatory response causes the pulmonary system to be less tolerant to the stressors of ischemia-reperfusion and subsequent mechanical insults [9]. Massive brain injury may precipitate ventilator induced lung injury, thereby worsening the outcome. This may occur through neurogenic pulmonary edema [10], ventilator associated pneumonia [11], and/or ALI/ARDS [12] that may result from inflammatory activation of pneumatocyte type II cells [13]. This may occur through the initiation and migration of activated neutrophils into the lungs [14]. In our institution there have been 264 patients with a GCS ≤ 8 over the past 36 months, 216 (81.8%) needed ventilator support (tracheal intubation), 64 (29.6%) acquired ARDS, and of these, 22/64 (34.3%) died.

Over the past 9 years inhaled nitric oxide (INO) has been used twice in a severely head injured human with ALI/ARDS with success [15,16]. INO, delivered at 10–80 parts per million (ppm), is a very effective pulmonary vasodilator [17] and improves arterial oxygenation [18-22]. However, INO use may not only improve arterial oxygenation, but it may also provide potent anti-inflammatory effects [23]. Here we report a case of severe traumatic brain injury with dramatically elevated intracranial pressure in the setting of ARDS that successfully responded to use of INO that was used as an adjunct to traditional therapy.

## Case presentation

**Figure 1:**
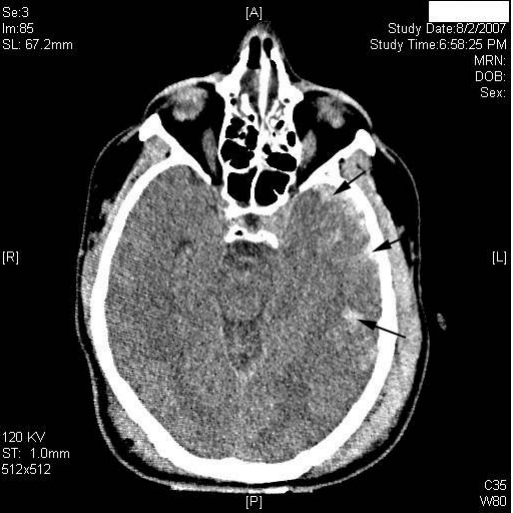
**Computed tomography scan at the level of the midbrain**. Multiple contusions involving the left temporal lobe are evident (arrows). A = anterior; P = posterior; L = left; R = right.

**Figure 2:**
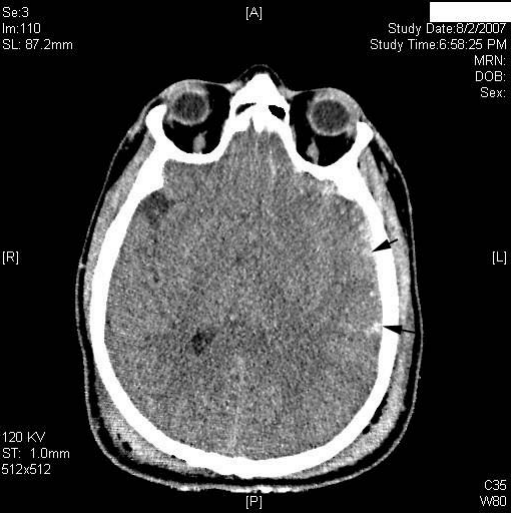
**Computed tomography scan at the level of the orbits**. Punctiform contusions involving the left temporal and frontal lobes with effacement of the left occipital horn are demonstrated (arrows). A = anterior; P = posterior; L = left; R = right.

**Figure 3:**
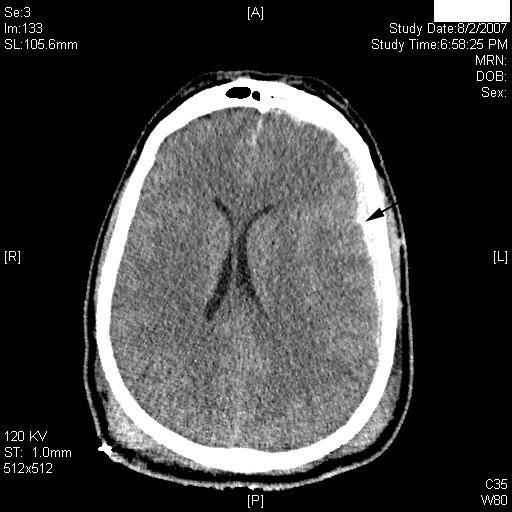
**Computed tomography scan at the level of the lateral ventricles**. There is a thin layer of acute subdural hematoma identified on the left with a minimal midline shift (arrow). A = anterior; P = posterior; L = left; R = right.

A 37 year old male involved in a motor cycle accident (unhelmeted) was found unresponsive at the scene. His trachea was intubated at the scene, resuscitated with fluids, and transported to the University of Toledo Medical Center via air ambulance. The patient arrived with a blood pressure 186/96 mm Hg, heart rate 79 beats per minute, SpO2 90% on 1.0 FiO2, and his Glasgow coma scale was 3. His pupils were round and equally reactive to light at 3 mm bilaterally. A right 9 French femoral venous sheath and a left femoral arterial catheter were placed on arrival. Complete blood count revealed hemoglobin 14.4 g/dl, hematocrit 41.8%, platelets 218,000/mm^3^, white blood cell count 19,800/mm^3^, sodium 143 meq/L, potassium 3.5 meq/L, chloride 109 meq/L, Carbon dioxide 26 mm Hg, blood urea nitrogen 12 mg/dl, creatinine 1.2 mg/dl, glucose 139 mg/dl, calcium 8.2 mg/dl, prothrombin time 15.8 seconds, international normalized ratio 1.2, albumin 3.4 g/dl, total bilirubin 0.9 mg/dl, alkaline phosphatase 64 IU/L, aspartate aminotransferase 57 IU/L, and alanine aminotransferase 43 IU/L. The initial arterial blood gas was pH 7.35, PaCO2 26 mm Hg, PaO2 60 mm Hg, HCO3 20 mmol/L, and base excess -5 mmol/L. The patient was given 10 mg vecuronium, and 25 grams of mannitol twice, intravenously. An electrocardiogram revealed a normal sinus rhythm, and the chest roentgenogram revealed bilateral pulmonary edema, a small left apical pneumothorax, multiple fractured ribs on the right, and a severely congested right lung field. His SpO2 suddenly decreased to 78% with diminished breath sounds bilaterally, and bilateral chest tubes were placed with improvement of the SpO2 to 90%. Subsequent computed tomography (CT) scan of the chest confirmed a left pneumothorax, that the right chest was filled with fluid (there was a question of aspiration), and the presence of bilateral pulmonary edema; CT of head demonstrated a left occipital fracture, multiple intraparenchymal hemorrhages, and a left subdural hematoma that was determined not to need surgical evacuation because of its size and minimal midline shift (see CT scan figures 1, 2, 3); and the CT scan of the abdomen was negative.

He was transferred to the intensive care unit where a right internal jugular 9 French sheath was placed for introduction of a pulmonary artery catheter, as was an intracranial pressure monitor (Camino). His initial ICP and cerebral perfusion pressure were 37 mm Hg and 57 mm Hg, respectively, on ventilator settings of assist control (AC) 30 (respiratory rate was 34), positive end expiratory pressure (PEEP) of 0 (zero) mm Hg, tidal volume (Vt) 650 ml, FiO2 1.0, with an ABG of pH 7.28, PaCO2 44 mm Hg, PaO2 63 mm Hg, and HCO3 20 meq/L. The SpO2 was 92%, the central venous pressure was 12 mm Hg, and the lactate was 8.5 mmol/L (which improved with further resuscitation).

Over the next seven days the patient's pulmonary status deteriorated and severe ARDS became manifest in the face of continued high ICP despite intensive intervention. On the morning of the seventh hospital day a critical point was reached. The ICP spiked to 70 mm Hg and remained over 50 mm Hg for greater than 2 minutes notwithstanding the use of mannitol, furosemide, hyperventilation, sedation, and paralysis, although these efforts maintained the CPP from 47–77 mm Hg. This high ICP occurred in the face of hypoxia and acidemia; ABG of pH 7.33, PaCO2 50 mm Hg, PaO2 54 mm Hg, base excess 0.1 mmol/L, and HCO3 26 meq/L while ventilated on AC 50 with Vt 500 ml, PEEP 7 cm H2O, and FiO2 1.0. The cardiac output was 11 liters/minute, and the cardiac index was 4.9 liters/minute/m^2^. The PEEP was raised to 17 cm H2O incrementally, while at the same time carefully evaluating ICP, peak inspiratory and plateau pressures, and oxygenation, in an effort to increase the PaO2 to an acceptable level (a goal of PaO2 100 mm Hg). In view of the fact that maximized ventilator settings, adequate sedation, paralysis, and inhalational therapies (albuterol and ipratropium) had neither improved the patient's intracranial pressures, nor his oxygenation, use of INO was implemented specifically to improve oxygenation and thereby decrease ICP. INO was instituted at 20 ppm. In a period of 35 minutes the ICP decreased to 15 mm Hg and PaO2 improved; ABG pH 7.35, PaCO2 49 mm Hg, PaO2 86 mm Hg, BE 0.7, and HCO3 27 meq/L on the same ventilator settings. Over 6 hours the PEEP was weaned to 11 cm H2O and the PaO2 remained at 90 mm Hg with the ICP ranging from 15–29 mm Hg. After 24 hours of INO at 20 ppm the ICP ranged from 12–20 mm Hg and the ABG was pH 7.47, PCO2 43 mm Hg, PaO2 165 mm Hg, BE 7.1 and HCO3 31 meq/L on AC 45, Vt 550, FiO2 95% and PEEP of 8 mm Hg. The INO was weaned over several days (there was no evidence of methemoglobinemia). The patient was discontinued from mechanical ventilation on hospital day 30. CT scan demonstrated no mass effect, but atrophy and hypodensity of the left temporal lobe. He was discharged to a rehabilitative traumatic brain injury unit on hospital day 34. Although he could follow commands, he had post-traumatic amnesia, a right hemiparesis, and moderate-severe cognitive, linguistic and language defects.

## Discussion

The patient's critically elevated ICP did not respond to traditional, aggressive neurointensive care modalities alone, but the addition of INO to these interventions was associated with a significant decrease in ICP that was life-saving. This patient's decrease in ICP could have occurred for several reasons. First, the occurrence of pulmonary vasodilation could have created a "sink" effect in which such vasodilation simply allowed more of the blood volume to remain in the thorax or to drain from the cerebral circulation to the thorax. Also, pulmonary vasodilation and increased PEEP (with an FiO2 of 1.0) could have provided improved oxygenation to the brain thus decreasing the ICP. In infants, however, there is evidence of increased cerebral blood flow with INO [24], thus potentially increasing ICP. Finally, while INO is a potent pulmonary vasodilator, and has been thought to remain only in the pulmonary system because it degrades quickly in vivo [17], it may act downstream (NO delivery beyond the lungs) to improve other organs through anti-inflammatory mechanisms [23]. The intriguing alternative of an anti-inflammatory extra-pulmonary delivery deserves further exploration.

The red blood cell (RBC) is now hypothesized to be the deliverer of nitric oxide (NO), not the consumer of NO [25]. NO reacts with heme iron and with cysteine (Cys)-93 on the hemoglobin β-sub unit [26]. NO reactions with heme iron will cause NO's inactivation, but S-nitrosylation of Cys-93 makes hemoglobin a carrier of NO bioactivity [27]. Also, an increase in S-nitrosothiol proteins occurs in sepsis (including RBC S-nitrosothio-hemoglobin and hemoglobin [Fe]NO) [28,29]. This accumulation of hemoglobin [Fe]NO as a 5-coordinate α-heme NO does not allow NO release to the Cys-β93 residue. Delivery of oxygen occurs without extensive vasodilation because of the dissociation of oxygen from the 5-coordinate α-heme-NO [30]. Thus, according to Goldfarb and Cinel, NO excess that interacts with hemoglobin will lead to products that prevent NO toxicity [31]. They also point out that S-nitrosylated albumin can transport NO bioactivity downstream to other organs [31] and that NO stabilized through hemoglobin, or other proteins through reversible S-nitrosylation, may be the manner in which NO extrapulmonary effects get downstream [31].

INO and glucocorticoid regulation is also of importance in sepsis and in TBI. Da et al demonstrated that glucocorticoid receptor (GR) up-regulation decreased the inflammatory response in a porcine model of sepsis using INO in combination with glucocorticoids (neither intervention worked well alone) [24]. However, contrary to Da et al, some animal models demonstrate that up-regulation of GR is neurotoxic [32-35]. After a cortical injury in these models, the rat hippocampus underwent cell loss because of an acute elevation of glucocorticoids. This model of GR up-regulation was considered detrimental [32]. A GR blocking agent, RU486 (mefepristone), though, was shown to be useful in this type of injury [34]. Therefore, there may be neuroprotection afforded to those with TBI due to down-regualtion by GR causing a low adrenocorticotropin (ACTH) level. This has been borne out in animals that underwent fluid percussion injury; their hypothalamic mRNA expression was increased [36]. High levels of total serum cortisol, ACTH, and catecholamines are present early in TBI [37,38], but a low plasma ACTH concentration in early TBI is associated with a better chance of intensive care unit (ICU) survival [39,40].

It may be that patients with TBI have adaptive down-regulation as demonstrated by Lee et al in an animal model in which cortical GR expression was down-regulated after 6 hours of injury in the ischemic cortex, and after 24 hours in the non-ischemic cortex in rats [41], indicating an attempt at neuroprotection. In humans such down-regulation has been demonstrated, but it may take a longer time [39]. Past studies have confirmed that the hippocampus is, indeed, involved in hypothalamic-pituitary-adrenal axis inhibition [42-44]. Stimulation of the hippocampus lowers glucocorticoid release in rats and humans [45,46], whereas hippocampal lesions increase corticosterone and corticotropin release in rats [47-50]. This probably occurs secondary to a damaged hippocampus, which will increase paraventricular corticotropin releasing hormone and arginine vasopressin gene expression resulting in an increase of ACTH [51-53].

INO reaching the central nervous system may allow GR in the brain to be downregulated. Aaltoren et al have shown that pigs with meconium aspiration have hippocampal neuronal injury [54]. However, when INO is administered to pigs with meconium aspiration, neuronal injury to the hippocampus is inhibited [55]. This occurs through diminished oxidation of DNA in the hippocampus and is accompanied by decreased levels of glutathione (a biomarker of oxidative stress) [55].

It is possible that INO moving downstream may be delivered to the brain and cause GR expression in the brain/hippocampus to be muted. This would produce a neuroprotective effect while at the same time allowing the rest of the body to up-regulate GR in response to steroids and INO administration thereby assisting the body in its efforts at combating inflammation.

The use of INO in this report was for emergent and compassionate use in the setting of critically elevated intracranial pressure accompanied by ARDS, hypoxemia, and high PEEP. The patient's spouse was fully informed of the reasons for the use of INO and the potential consequences. The institutional ethics committee was not consulted for use of INO in this situation because of the compressed time-line for decision-making and intervention. However, after the event members of the ethics committee and the institutional review board were consulted regarding the continued use of INO in this case and similar situations in the future.

## Conclusion

INO in humans with TBI and ARDS has now been used successfully on three occasions to improve outcomes. Although it has also been shown to be effective in hippocampal preservation and in decreasing inflammation in animals, any hypothesis regarding humans arising from our observations should be tested in rigorously designed experimental and clinical studies.

## Consent

The corresponding author received consent from the patient's wife (next of kin) for publication of this report.

## Competing interests

The authors declare that they have no competing interests.

## Authors' contributions

TJP cared for the patient and participated in all portions of the paper, AM cared for the patient and participated in the case report section, SY participated in the case report section.
